# EXPERIENCES OF INNER STRENGTH AT THE TIME OF MILD COGNITIVE IMPAIRMENT DIAGNOSIS

**DOI:** 10.1093/geroni/igac059.1006

**Published:** 2022-12-20

**Authors:** Brianna Morgan, Lauren Massimo, Sharon Ravitch, Jason Karlawish, Nancy Hodgson

**Affiliations:** University of Pennsylvania, Philadelphia, Pennsylvania, United States; University of Pennsylvania, Philadelphia, Pennsylvania, United States; University of Pennsylvania, Philadelphia, Pennsylvania, United States; University of Pennsylvania Perelman School of Medicine, Philadelphia, Pennsylvania, United States; University of Pennsylvania, Philadelphia, Pennsylvania, United States

## Abstract

Inner strength is a person’s internal process of moving through challenging circumstances, such as receiving a diagnosis of Mild Cognitive Impairment (MCI). This study describes experiences of inner strength using qualitative methodologies to identify themes within semi-structured dyadic and individual interviews with persons diagnosed with MCI within 12 months at a Memory Center and their care partners. We analyzed data in NVivo using reflexive thematic analysis methods. Trustworthiness was maintained through vetted interview guides, verbatim transcription, field notes, peer group analysis, and audit trails. One overarching theme and three subthemes explained inner strength. An overarching theme, Finding Ways to Live with It, described how participants live within the circumstances of MCI. Three subthemes were Defining Strength by Recalling the Past, Seeking Relief and Dwelling in It, and Finding Purpose & Meaning. Implications include supporting inner strength at the time of MCI diagnosis through reminiscence therapy and meaning making interventions.

